# A simple and efficient strategy for trace detection of ferroptosis-related miRNAs based on novel hydrophobic paper-based plasmonic substrate and “inverse molecular sentinel (iMS)” nanoprobes

**DOI:** 10.3389/fbioe.2023.1146111

**Published:** 2023-03-02

**Authors:** Youwei Wang, Bing Chen, Jiang Fan, Zhong Wang

**Affiliations:** ^1^ Department of neurosurgery, The First Affiliated Hospital of Soochow University, Suzhou, China; ^2^ Department of neurosurgery, The Affiliated Hospital of Yangzhou University, Yangzhou, China; ^3^ Department of neurosurgery, The Affiliated hospital of Qingdao University, Qingdao, China; ^4^ Department of neurosurgery and Brain and Nerve Research Laboratory, The First Affiliated Hospital of Soochow University, Suzhou, China

**Keywords:** surface-enhanced Raman scattering, miRNA, microRNA, inverse molecular sentinel, intracerebral hemorrhage (ICH), dumbbell-shaped gold nanorods

## Abstract

Monitoring ferroptosis-related miRNAs is crucial for the treatment and prognosis of patients with intracerebral hemorrhage. In this work, a novel hydrophobic paper (h-paper)-based plasmonic substrate was produced by dropping DS Au nanorods with a narrow range of sizes and morphologies onto h-paper. Raman reporter molecules were adsorbed to the array surface, and surface-enhanced Raman scattering spectra at randomly selected points reveal uniform and significant SERS enhancement. Hairpin DNAs labelled with Raman reporters and hybridized with placeholder DNAs were decorated on SERS substrate to fabricate SERS biosensor. Target miRNAs initiated the “inverse Molecular Sentinel” process. During the process, PHs were removed and the conformation of HPs changed toward the hairpin structure, thus eliciting the proximity of Raman reporter to substrate and a stronger SERS signal. The proposed SERS biosensor performs well in terms of stability, reproducibility, and selectivity. The limits of detection of miR-122-5p and miR-140-5p in serum were 4.17 aM and 4.49 aM, respectively. Finally, the fabricated SERS biosensor was applied to detect miR-122-5p and miR-140-5p in ICH patients and healthy subjects, and the results obtained by SERS were consistent with the results from quantitative real-time polymerase chain reaction, revealing the accuracy of the method. This simple, rapid approach offers great potential for the simultaneous detection of miRNAs in practical clinical applications.

## 1 Introduction

Intracerebral hemorrhage (ICH) is a disastrous disease with high rates of mortality and morbidity, accounting for 10%–15% of all stroke types (Zhou et al., 2020; Han et al., 2021). In the early stage of ICH, blood seeps into the brain causing acute intracranial hypertension. As the disease progresses, the hemoglobin (Hb) released from lysed red blood cells is phagocytized by phagocytes and metabolized into iron (Wan et al., 2019), which is responsible for ferroptosis (Li et al., 2017; Han et al., 2021). Ferroptosis, a form of programmed cell death, is identified with the iron-dependent excessive accumulation of lethal lipid peroxidation (Yin et al., 2022). Emerging data have shown that ferroptosis is involved in the secondary injury, and contributed to blood-brain barrier (BBB) disruption and neurological deficits after ICH (Zhang et al., 2018a; Chen et al., 2019a; Wei et al., 2022). Ferrostatin-1, as a ferroptosis inhibitor, can reduce Hb-induced death of primary cortical neurons by more than 80% *in vitro* (Zille et al., 2017) and display neuroprotective effect through reducing iron deposition in the perihematomal brain tissues (Chen et al., 2019a). Interventions against ferroptosis are important strategies for the treatment of brain injury secondary to ICH. Thus, it is crucial to monitor biomarkers of ferroptosis in ICH in real time. MicroRNAs (miRNAs) are endogenous non-coding single-stranded RNA molecules with lengths ranging from 21 to 25 nucleotides, which regulate various metabolic pathways by targeting messenger RNA (mRNA) to inhibit the post-transcriptional and translational levels (Dai et al., 2022; Guo et al., 2022). Studies have reported that miRNAs play a crucial role in the ferroptosis of various diseases, such as tumors (Dai et al., 2022), heart failure (Zheng et al., 2021), traumatic brain injury (Wu et al., 2022), as well as ICH. Further investigation indicated that higher level of serum miRNAs were correlated with poorer neurologic scores of ICH patients (Sun et al., 2020a; Bao et al., 2020; Wan et al., 2021; Yin et al., 2022). Yin M et al. proposed that overexpressed miR-140-5p attenuates ICH-induced brain injury and neuroinflammation by inhibiting neuronal ionization *via* the miR-140-5p/GSK-3β axis (Wang et al., 2019). Zhao HK et al. introduced isorhynchophylline, which had strong antioxidant activity, to investigate its effect on ferroptosis after ICH. They concluded that isorhynchophylline could alleviate ferroptosis-induced neurological damage following ICH by activating miR-122-5p, and a high level of miR-122-5p was associated with reduced ferroptosis (Zhao et al., 2021a). Thus, miRNAs may be used as reliable biomarkers to track ferroptosis following ICH. So far, numerous conventional techniques, including RT-PCR, microarrays, northern blotting, and so on have been used to detect miRNAs (Koscianska et al., 2011; Wu et al., 2013; Sanchez et al., 2018). However, each of these methods has its own shortfalls, such as time-consuming, elaborate, and expensive laboratory equipment. So, an alternative detection strategy that can simply, rapidly, and precisely detect miRNAs associated with ferroptosis following ICH is urgently needed. As far as we know, employing other techniques for detecting ferroptosis-related miRNAs has not been reported.

Surface-enhanced Raman spectroscopy (SERS) has received much attention in the field of biomarkers analysis. SERS is a physical phenomenon that enhances Raman scattering when a laser excites specific rough noble metal nanoparticles. SERS technology is an analysis method based on this, which can greatly improve the detection sensitivity (Li et al., 2016; Wee et al., 2016; Qiu et al., 2022). The technology exhibits rapid, fingerprint identification and ultra-sensitive characteristics, and has been cited extensively in many fields, especially in biological analysis and life sciences (Sun et al., 2020b; Ge et al., 2022a; Ge et al., 2022b). The ultra-sensitivity of SERS relies prominently on the localized surface plasmon resonance (LSPR) effect on the surface of metal nanostructures, which is mainly attributed to nanomaterial physicochemical properties, including size, shape, as well as material composition. Thus, the selection of a suitable substrate is a very influential factor for SERS. In recent years, gold nanorod has attracted enormous attention due to its tunable LSPR in the near-infrared visible band, unique optical properties, and good biocompatibility (Jeon et al., 2016; Zhao et al., 2021b; Sau and Murphy, 2004; Murphy et al., 2010). In order to obtain greater enhancement ability, gold nanorods have been elaborately designed into various shapes, including biconical gold nanorods with greater local electric field enhancement ability (Jeon et al., 2016), and dumbbell-like gold nanorods with both tunable LSPR and greater local electric field enhancement (Chapagain et al., 2021). The surface morphology of gold nanorods also plays an extremely significant role in Raman enhancement. Generally speaking, rough and irregular nanoparticles have greater enhancement ability than smooth ones (Mei et al., 2020). Novel dumbbell-shaped gold nanorods (DS Au nanorods) combine the superior properties of gold nanorods and biconical nanorods, exhibiting excellent SERS performance and especially for SERS sensing technology (Jeon et al., 2016).

Cerebrospinal fluid (CSF) is considered to be the most valuable specimen for the identification of ICH biomarkers. However, some brain-derived diseases such as stoke and traumatic brain injury can lead to the disruption of BBB, resulting in the release of relevant biomarkers into the peripheral blood (Jiang et al., 2018; Gao et al., 2021). Detection of miRNAs in peripheral blood avoid the risk and complexity of lumbar puncture. In literatures, various SERS-based analytical methods were developed for miRNAs detection, which were classified into label-free and label-based methods. The label-free approach relied on the precise detection of miRNA’s intrinsic Raman vibration. However, it was difficult to identify a specific miRNA sequence due to the high sequence homology of miRNAs (Cao et al., 2002). To further enhance the detection sensitivity and specificity, label-based strategies were introduced, including the rolling circle amplification (RCA), duplex-specific nuclease (DSN), hybridization chain reaction (HCR), catalytic hairpin assembly (CHA), etc. RCA and DSN were enzymatic amplification strategies, which had the disadvantages of complicated operation process, susceptibility to biological environment, and reaction time depending on enzyme activity (Cao et al., 2022). HCR and CHA were powerful signal amplification strategies with enzyme-free reactions. However, the usage of two probes had the shortcomings of a sophisticated labeling procedure, time-consuming, and high cost of reagents (Chen et al., 2019b). Furthermore, the non-specific products, which were produced in the absence of target, generated a significant amount of background noise and reduced the effectiveness of amplification. Recently, the novel “inverse Molecular Sentinel (iMS)” achieved simple, fast, and accurate detection of miRNAs. In iMS, specific target identification and signal switch employed a nonenzymatic DNA strand-displacement process (Nie and Emory, 1997). Additionally, the enhancement of Raman scattering from iMS could be increased by an enhancement factor of 10^6^–10^7^, or even achieved as large as 10^15^ at “hot spots” and allowed the detection of single molecules (Wang et al., 2016).

Herein, a novel SERS biosensor based on hydrophobic paper (h-paper)-based plasmonic substrate and iMS was designed for simultaneous detection of miR-122-5p and miR-140-5p associated with ferroptosis following ICH. Firstly, the h-paper was prepared by soaking the filter paper in an alkyl ketene dimer (AKD) solution to convert the hydrophilic hydroxyl groups of the cellulose fibers in the paper into hydrophobic alkyl groups. The h-paper could prevent analytes and nanoparticles from rapidly absorbing, improving uniformity and reproducibility (Lee et al., 2018). Then, the plasmonic DS Au nanorods with a narrow distribution of sizes and morphologies were dropped onto h-paper to fabricate the h-paper-based plasmonic substrate. The SERS biosensor was prepared by modifying DS Au nanorods array with hairpin DNAs (HPs), which were labeled with Cy5 (or 5-FAM) and partly hybridized with single-stranded DNAs named “placeholder DNAs” (PHs). The hybridization between HP and PH opened the hairpin structure in HP and formed a linear double-stranded DNA duplex, which compelled Raman reporters away from the substrate, showing “OFF” status with a weak Raman signal. When miR-122-5p (and miR-140-5p) was present, the iMS process was initiated: target miRNAs bound specifically to the PHs due to the stronger binding affinity, which initiated a nonenzymatic DNA strand-displacement process and formed complementary DNA double chains, leading HPs gradually transforms into hairpin structures. By this way, Raman reporters closed to the substrate and SERS biosensor exhibiting “ON” status with a strong Raman signal. Scanning electron microscopy (SEM), and SERS spectra were performed to evaluate substrate homogeneity and stability. Optimization of several key experimental parameters was performed to fabricate ultra-sensitive biosensor. Additionally, the reproducibility, stability, uniformity, and specificity of the substrate were assessed, followed by the SERS analysis of miR-122-5p and miR-140-5p in serum simultaneously. Eventually, this approach was applied for target miRNAs detection in practical samples and its accuracy was verified by qRT-PCR, demonstrating the potential application of SERS-based biosensor for ferroptosis following ICH.

## 2 Materials and methods

### 2.1 Materials and reagents

Chloroauric acid tetrahydrate (HAuCl_4_), silver nitrate (AgNO_3_), sodium borohydride (NaBH_4_), ascorbic acid (AA), trisodium citrate dihydrate (TSC), cetyltrimethylammonium chloride (CTAC), benzyldimethylammoniumchloride hydrate (BDAC), concentrated nitric acid (HNO_3_; 67%), hydrochloric acid (HCl; 37%), sodium hydroxide (NaOH), cyclohexane and absolute ethanol were obtained from Yangzhou Feichang Chemicals Co. Ltd. (China). Phosphate buffer saline (PBS), 5-carboxyfluorescein (5-FAM), Cyanine5 (Cy5) were obtained from Shanghai Aladdin Biochemical Technology Co., Ltd. alkyl ketene dimer (AKD) and filter paper were purchased from Younuo Chemicals Inc. (Yangzhou, China). All oligonucleotide sequences in Supplementary Table S1 including miR-122-5p, miR-140-5p, HP1, HP2, PH1, PH2, one-base mismatch sequences (MT1-1 and MT1-2), three-base mismatch sequences (MT3-1 and MT3-2) and random sequences were acquired from Sangon Biotech Co. Ltd. (China). All reagents used in the experiment were analytically pure, and ultra-pure water (Milli-Q, Millipore Corp.) was used in all experiments.

### 2.2 Clinical samples collection

Human peripheral blood samples were kindly provided by healthy volunteers and the Affiliated Hospital of Yangzhou University and the Affiliated Hospital of Qingdao University including 40 patients with ICH. All subjects were informed of the goals of the study and gave their written consent to participate. Human peripheral blood samples were kindly provided by healthy volunteers, the Affiliated Hospital of Yangzhou University and the Affiliated Hospital of Qingdao University (Supplementary Table S2). Blood (5 mL) was drawn and collected in EDTA tubes, followed immediately by centrifugation at 12,000 rpm for 10 min (4°C). The separated supernatants were collected after centrifugation and stored at −80°C for subsequent experiments.

### 2.3 Synthesis of DS Au nanorods

DS Au nanorods were prepared by seed-mediated method. Firstly, the preparation of decahedral Au nanoseeds followed the previously reported experimental protocol (Sanchez-Iglesias et al., 2017) with some modifications: aqueous solutions of HAuCl_4_ (0.5 mM, 3.56 mL) and CTAC (200 mM, 2.5 mL) were sequentially added to deionized water (1.15 mL), and the solution was stirred magnetically at room temperature for 30 min. Then trisodium citrate solution (20 mM, 2.5 mL) and newly prepared NaBH_4_ solution (25 mM, 0.25 mL) were added successively under vigorous stirring for 2 min. The reaction vessel was transferred to an oil bath at 85°C and stirred gently for 4.5 h, the color gradually changed from brown to wine red, indicating the formation of decahedral Au nanoseeds. The prepared seed solution was removed from the bath and stored at room temperature. Secondly, a growth solution containing BDAC (93 mM, 9 mL) and HAuCl_4_ (10 mM, 0.35 mL) was immersed in a thermostatic bath at 30°C under constant stirring. 30 min later, 200 μL HCl (1 M) and 100 μL AgNO_3_ (10 mM) were added to the mixture, followed by stirring for 1 min at 450 rpm. Then, 75 μL AA (100 mM) and 100 μL as-prepared decahedral Au nanoseeds solution was introduced and left in a water bath at 30°C for 2 h after stirring for 1 min. After the reaction, the resulting solution was centrifuged at 9,000 rpm for 12 min, washed twice with ultrapure water, and dispersed in CTAC solution for further use.

### 2.4 Fabrication of SERS substrate

At first, the h-paper was performed by soaking the hydrophobic filter paper in a 0.1% AKD dispersion for 10 min, followed by placed in an oven at 100°C (Sun et al., 2020a). The fabrication of SERS substrate was divided into two steps: assembly and functionalization. Firstly, 2 ml of DS gold nanorod solution and 4 mL of cyclohexane were added to a 20 mL beaker and stirred thoroughly for 3 min. After adding absolute ethanol dropwise and standing for 5 min, the fabrication of DS Au nanorods arrays was completed at the interface of oil and water by self-assembly. Secondly, the monolayer array formed at the oil-water interface was transferred onto the h-paper by pipettor and then dried. The following step was the surface functionalization of the DS Au nanorods array by decorating HPs and PHs. HPs and PHs were activated by placing them in a water bath above melting temperature (Tm), referring to the primer melting temperature, and were calculated by the corporation of Minneapolis. Next, the array was incubated with 100 μL of the mixed solution including 1 μM HP1 and an aliquot of HP2 for 80 min. HP1 and HP2 can be attached on the surface of DS Au nanorod *via* Au-S bonds. The substrate was washed three times with ultrapure water to remove excess HP1 and HP2. Then the substrate was incubated in the solution mixed with PH1 (50 μL, 1 μM) and PH2 (50 μL, 1 μM) at 37°C for 50 min to complete the hybridization. Finally, excess unreacted reagent on the above-functionalized substrate was washed off twice with PBS and then dried at room temperature for SERS measurements.

### 2.5 Procedure of detecting target miRNAs

Target miRNAs solution (10 μL) with different concentrations was added separately to the aforementioned SERS biosensor and incubated in a humid chamber at 37°C for 120 min. Spectra were collected after thoroughly washing substrate with PBS. The laser wavelength was 785 nm, the power was 5 mW, and the exposure time was 10 s. Each final value is the average spectral value obtained by repeating the experiment three times at three different locations to ensure the accuracy and representativeness of the results. The spectra were collected from 800 to 1800 cm^−1^ with a spectral resolution of 3 cm^−1^. All data were analyzed with SPSS Statistics 21.0 (Chicago, IL, United States). When the *p*-value was less than 0.05 (*p* < 0.05), the difference was considered statistically significant.

### 2.6 Characterization

All Raman spectra were measured with a Renishaw (in Via) Raman microscope spectrometer (785 nm excitation) with a ×50 objective lens. The WiRETM software of Renishaw was used for Raman system operation and data acquisition. To repress the background noises of instrument, smoothing and baseline correction were applied. The UV-visible absorption spectra were obtained from a UNICO 2100 PC UV-Vis spectrophotometer and processed with Origin Lab software. Transmission electron microscopy (TEM) images of DS Au nanorods were viewed and characterized under a Tecnai 12 transmission electron microscope (Philips) at an accelerating voltage of 60 kV. Scanning electron microscopy (SEM) images of DS Au nanorods were observed using a field-emission scanning electron microscope (Hitachi, S-4800 Ⅱ) at a 1 kV accelerating voltage. The high-resolution TEM (HRTEM) was captured with a Tecnai G2 F30 S-Twin TEM (FEI) at 200 kV.

## 3 Results and discussion

### 3.1 Principle of simultaneous detection of target miRNAs

**FIGURE 1:**
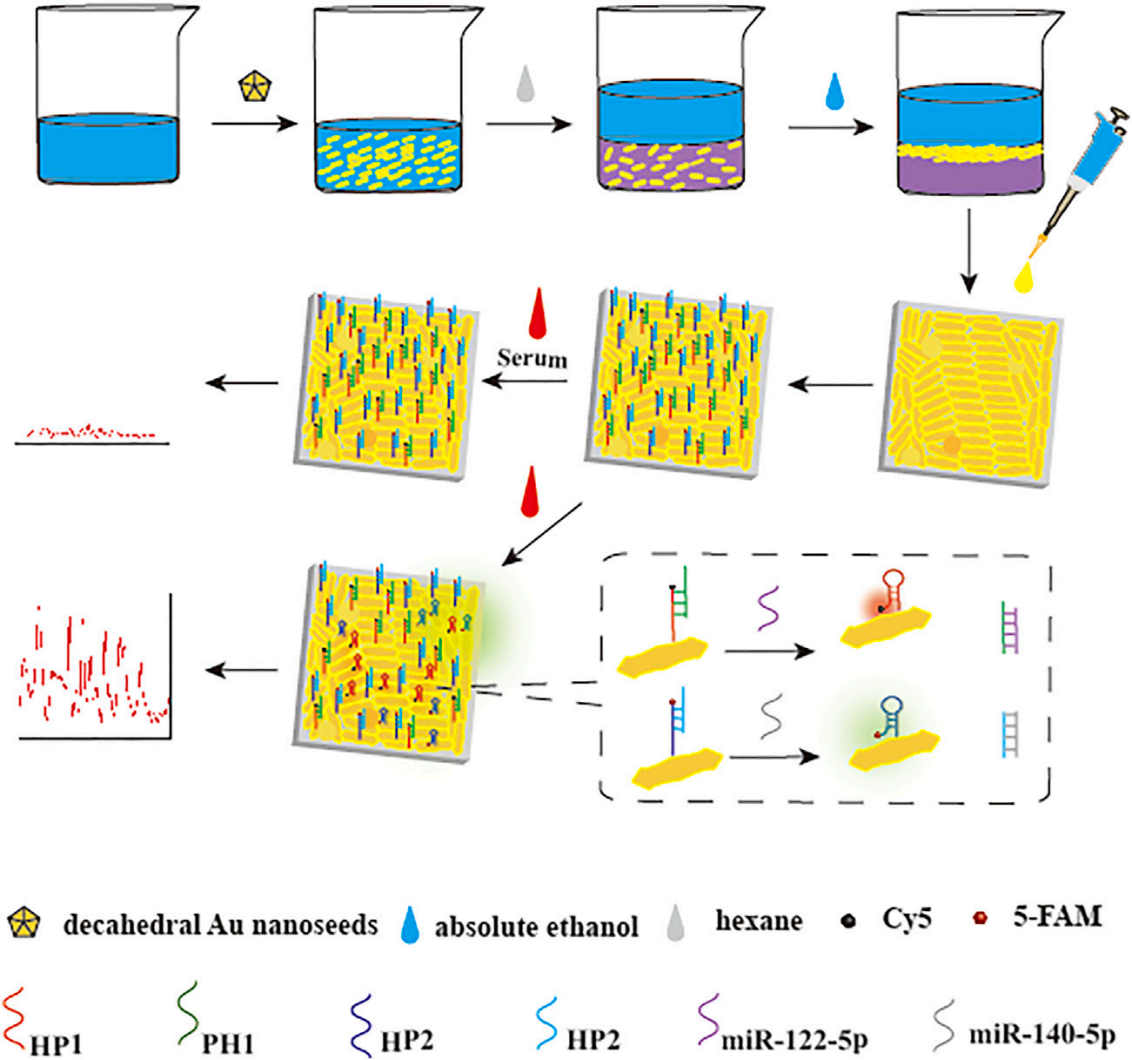
Principle of simultaneous detection of target miRNAs. SERS biosensor was fabricated by decorating DS Au nanorods-modified h-paper with HPs, which were hybridized with PHs and labeled with Raman reporters. In the presence of target miRNAs, the “inverse Molecular Sentinel” (iMS) process initiated and induced HPs the conformation change toward the hairpin structure. The hairpin structure of HPs results in the close proximity of Raman reporters to the substrate and a stronger SERS signal.

The SERS assay strategy for miR-122-5p and miR-140-5p detection was shown in Figure 1. The first preparatory work was the construction of substrate: the DS Au nanorods array was transferred from the interface of oil and water onto the h-paper by pipettor and then dried. The next step was the *in-situ* functionalization of the substrate. Due to the presence of chain sulfhydryl groups, HP1 and HP2 could be efficiently adsorbed on the DS Au nanorod surface. PH1 and PH2 were then linked to the complementary DNA (HP1 or HP2) by hybridization effects. After the above steps, the SERS biosensor can be successfully prepared. Subsequently, after the addition of target miRNAs, the SERS biosensors could capture and hybridize with the target miRNAs, resulting in the detachment of PHs and the formation of PH/RNA heteroduplexes. The remaining HPs reverted to hairpin structure, and the SERS signal gradually increased as the distance between the Raman reporters and the substrate decreased. Based on the above mechanism, the target miRNAs in clinical serum can be quantitatively detected through the change of SERS signal intensity.

### 3.2 Characterization of DS Au nanorods

**FIGURE 2:**
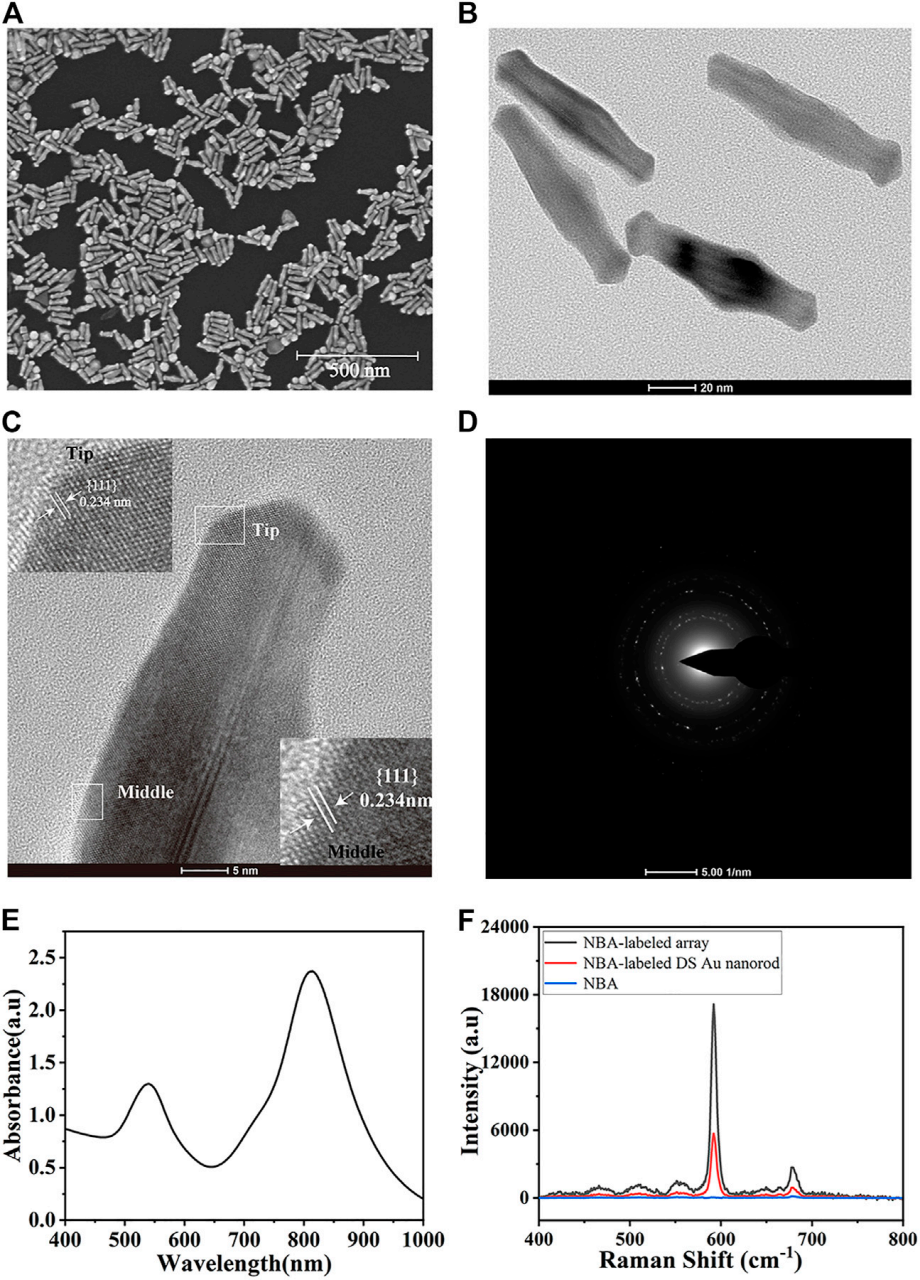
**(A)** SEM image and **(B)** TEM image of DS Au nanorods. **(C)** HRTEM image and magnified images of lattice fringes at the tip and middle of DS Au nanorod. **(D)** SAED pattern of DS Au nanorod. **(E)** UV-vis absorption spectrum of DS Au nanorod. **(F)** SERS spectra of pure NBA-labeled DS Au nanorod.

The physical properties such as morphology and size of the as-prepared DS Au nanorods were analyzed using SEM and TEM images. As shown in Figure 2A, DS Au nanorods maintained uniform in both size and morphology. The typical TEM image (Figure 2B) indicated that all of them are dumbbell-shaped with a longitudinal size of about 88 nm and a transverse size of 24 nm. Figure 2C is a high-resolution TEM (HRTEM) image used to determine the crystal structure of DS Au nanorods. Figure 2C presented clear lattice fringes with an interplanar spacing of 0.234 nm, corresponding to the {111} planes of Au. The magnified images in Figure 2C show clear lattice fringes at the tip and middle of the particles. In addition, a SAED image (Figure 2D) was employed to further observe the morphology of DS Au nanorods. The UV-vis-NIR absorption spectrum of DS Au nanorods was shown in Figure 2E, showing a major LSPR band centered at 810 nm and a less intense and broader band at 540 nm. Figure 2F depicted the SERS spectra of NBA at a concentration of 10^–2^ M, NBA-labeled DS Au nanorods (10^–6^ M), and NBA-labeled monolayer DS Au nanorod arrays for investigating the enhancement effect of DS Au nanorods. As can be seen from Figure 2F, pure NBA exhibited almost no SERS signal in the absence of DS Au nanorods, NBA-labelled DS Au nanorods showed a strong SERS signal. Furthermore, the SERS signal of NBA-labeled monolayer DS Au nanorods array was stronger than that of NBA-labelled DS Au nanorods. This indicates that DS Au nanorods exhibited excellent SERS enhancement effect and the SERS enhancement of DS Au nanorods arrays is significantly higher than that of DS Au nanorod alone. The enhancement effect of DS Au nanorods was quantified by the enhancement factor (EF), which was calculated by the following formula (Su et al., 2019): EF = (I_SERS_/C_SERS_)/(I_RS_/C_RS_), where I_SERS_ was the SERS signal intensity obtained for the DS Au nanorods at a specific concentration (C_SERS_), whereas C_RS_ was the concentration of the NBA, which produced a Raman signal I_RS_ under non-SERS conditions. Thus, the EF value of DS Au nanorod was 5.3×10^7^ when C_SERS_ was set to 10^–6^ M and C_RS_ was 10^–2^ M. Besides, the EF values of the monolayer DS Au nanorod array was calculated and the value is 4.6×10^8^, which is 1 order of magnitude higher than that of the DS Au nanorods solution, indicating that the DS Au nanorods array can provide significantly enhanced SERS effect.

### 3.3 Characterization of SERS substrates

**FIGURE 3:**
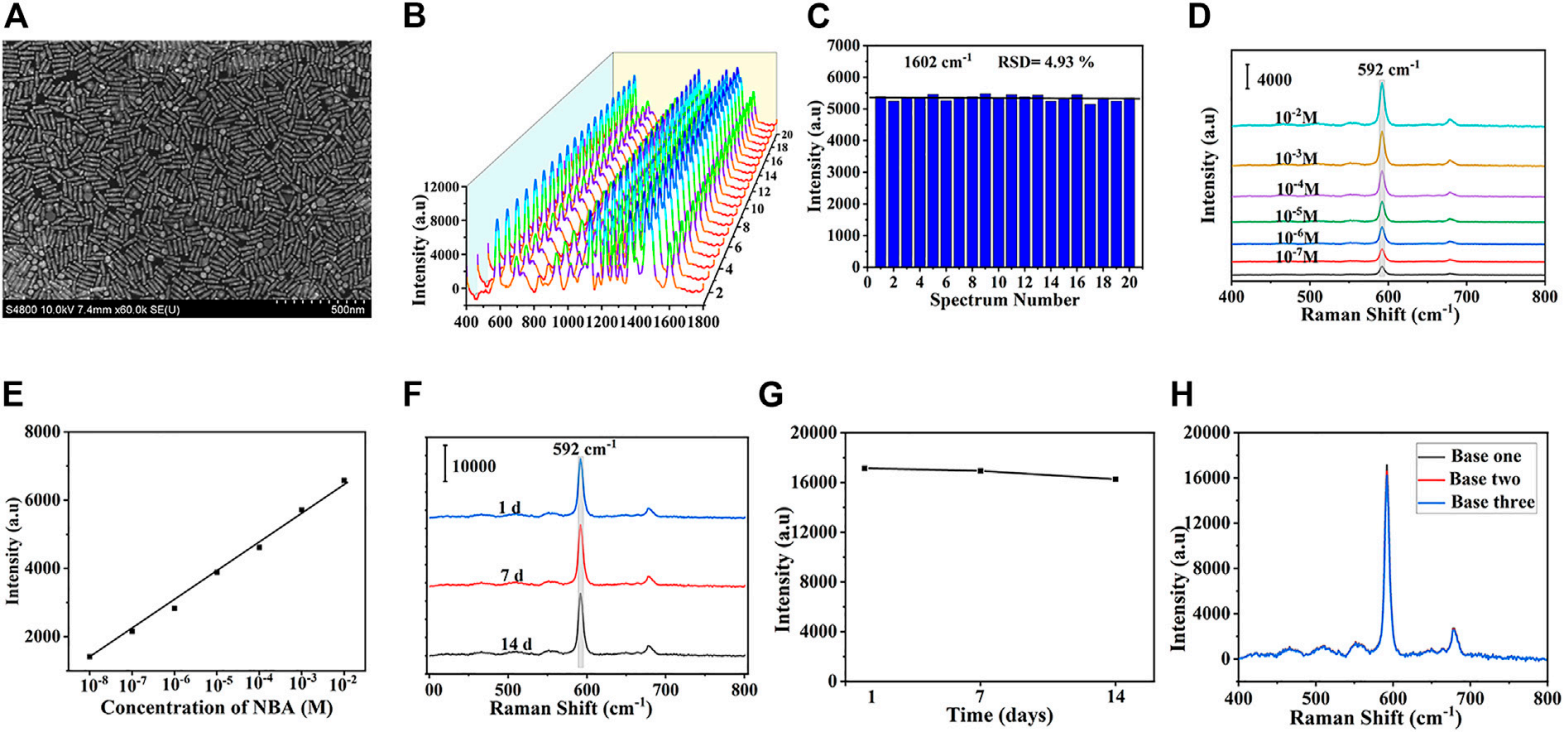
**(A)** SEM images of DS Au nanorod array. **(B)** SERS spectra at 20 randomly selected spots and **(C)** the corresponding signal intensity histogram at 1,062 cm^−1^. **(D)** SERS spectra of DS Au nanorod array at different concentrations of NBA (10^−2^-10^−8^ mol/L). **(E)** Relationship between SERS intensity and logarithm of NBA concentration at 592 cm^−1^. **(F)** The average SERS spectra of the NBA-labeled SERS substrate after the storage of 1 day, 7 days, 14 days and **(G)** the corresponding SERS intensities of the bands at 592 cm^−1^. **(H)** SERS spectra of three NBA-labeled DS Au nanorod array synthesized at different batches.

The uniformity, sensitivity, and stability of SERS substrate are the key to SERS detection. The SEM image of the substrate was shown in Figure 3A, which showed that DS gold nanorods are uniformly distributed with only minor aggregation. In order to further explore the uniformity of the substrate, a mixed solution of 5-FAM and Cy5 (10^–6^ mol/L) was adsorbed on the surface of SERS substrate, then 20 spots were randomly selected and SERS spectra were performed with the intensity of the characteristic peak at 1,602 cm^−1^, which were presented in Figure 3B. The corresponding signal intensity histogram at 1,062 cm^−1^ was depicted in Figure 3C with a relative standard deviation (RSD) of 4.93%, indicating the excellent uniformity of SERS substrate. To assess the sensitivity of the substrate, SERS assays were performed on a series of different concentrations of NBA. NBA was chosen as the Raman report molecule due to its distinct Raman properties and ability to self-assemble a monolayer on gold nanoparticles without further chemical modification. The characteristic peak of NBA at 592 cm^−1^ formed by the positively charged nitrogen was applied (Bi et al., 2013). Figure 3D depicted the SERS Raman spectra of NBA at various concentrations, revealing that the Raman enhancement of signal molecules increases with the concentration of NBA. Figure 3E showed the linear relationship between various concentrations of NBA and SERS intensities, where the horizontal axis represented the NBA concentration, and the vertical axis stood for the SERS intensity at 592cm^−1^. A satisfactory linear relationship was exhibited, and the detection range of NBA can be performed in the range of 10^–2^ to 10^–8^ M. In addition to verifying the uniformity and sensitivity of the substrate, the stability of the substrate was one of the critical properties for a SERS biosensor. Figure 3F displayed the average SERS spectra of the NBA-labelled SERS substrate after storage for 1 day, 7 days, and 14 days at room temperature. There was no obvious change in the intensity and shape of the SERS spectrum, which indicated that the substrate achieved great stability. In Figure 3G, the SERS enhancement effect of the substrate after 14 days of storage at room temperature was only reduced by 5.13% compared with the freshly prepared one, further proving the SERS stability of the substrate. At last, The SERS spectra of three NBA-labelled SERS substrates produced at three distinct periods were measured. The variance of the signal intensity at 1,602 cm^−1^ was 5.38%, as shown in Figure 3H, suggesting that the SERS substrate was reproducible.

### 3.4 Evaluation of cross-reactivity

**FIGURE 4:**
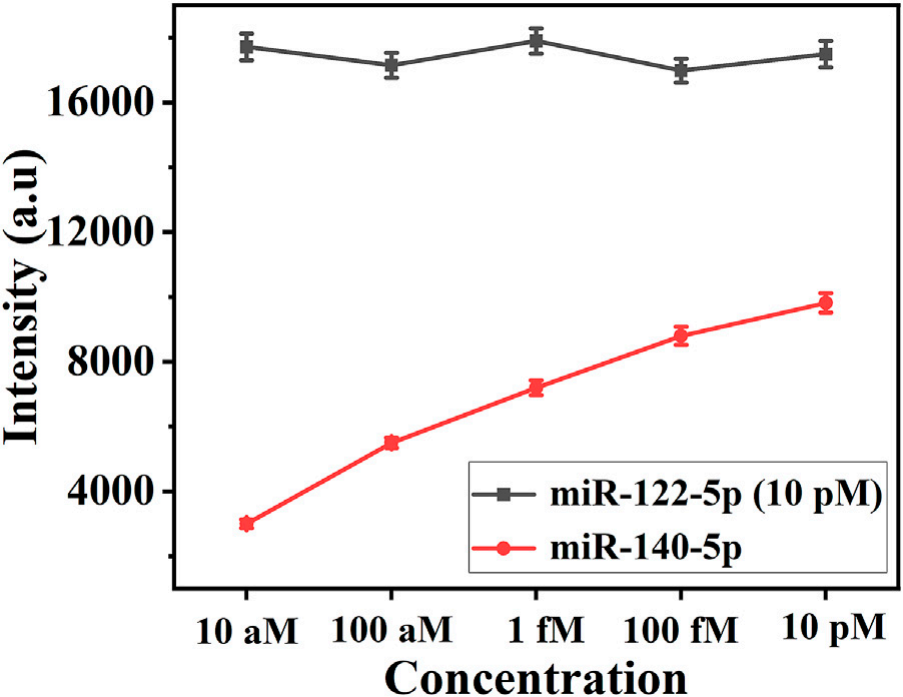
Line chart of SERS intensities at 1,133 cm^−1^ and 1,602 cm^−1^. The concentration: (1) 10 pM miR-122-5p +10 aM miR-140-5p; (2) 10 pM miR-122-5p +1 fM miR-140-5p; (3) 10 pM miR-122-5p +100 fM miR-140-5p; (4) 10 pM miR-122-5p +1 pM miR-140-5p; (5) 10 pM miR-122-5p +10 pM miR-140-5p.

Determining whether there is a cross-reaction between miR-122-5p and miR-140-5p is the key to the simultaneous analysis of two miRNAs in this experiment. 10 pM of miR-122-5p was mixed with miR-140-5p at concentrations ranging from 10 aM to 10 pM for evaluating cross-reactivity between the two miRNAs. In this work, the characteristic Raman peak intensities at 1,133 cm^−1^ (5-FAM) and 1,602 cm^−1^ (Cy5) were used for the quantitative detection of miR-122-5p and miR-140-5p, respectively. As depicted in Figure 4, the intensity of the SERS signal at 1,602 cm^−1^ increased linearly as the concentration of miR-140-5p increased, whereas the intensity at 1,133 cm^−1^ remained constant. The result demonstrated the feasibility of detecting the two miRNAs simultaneously.

### 3.5 Optimization of experimental parameters

**FIGURE 5:**
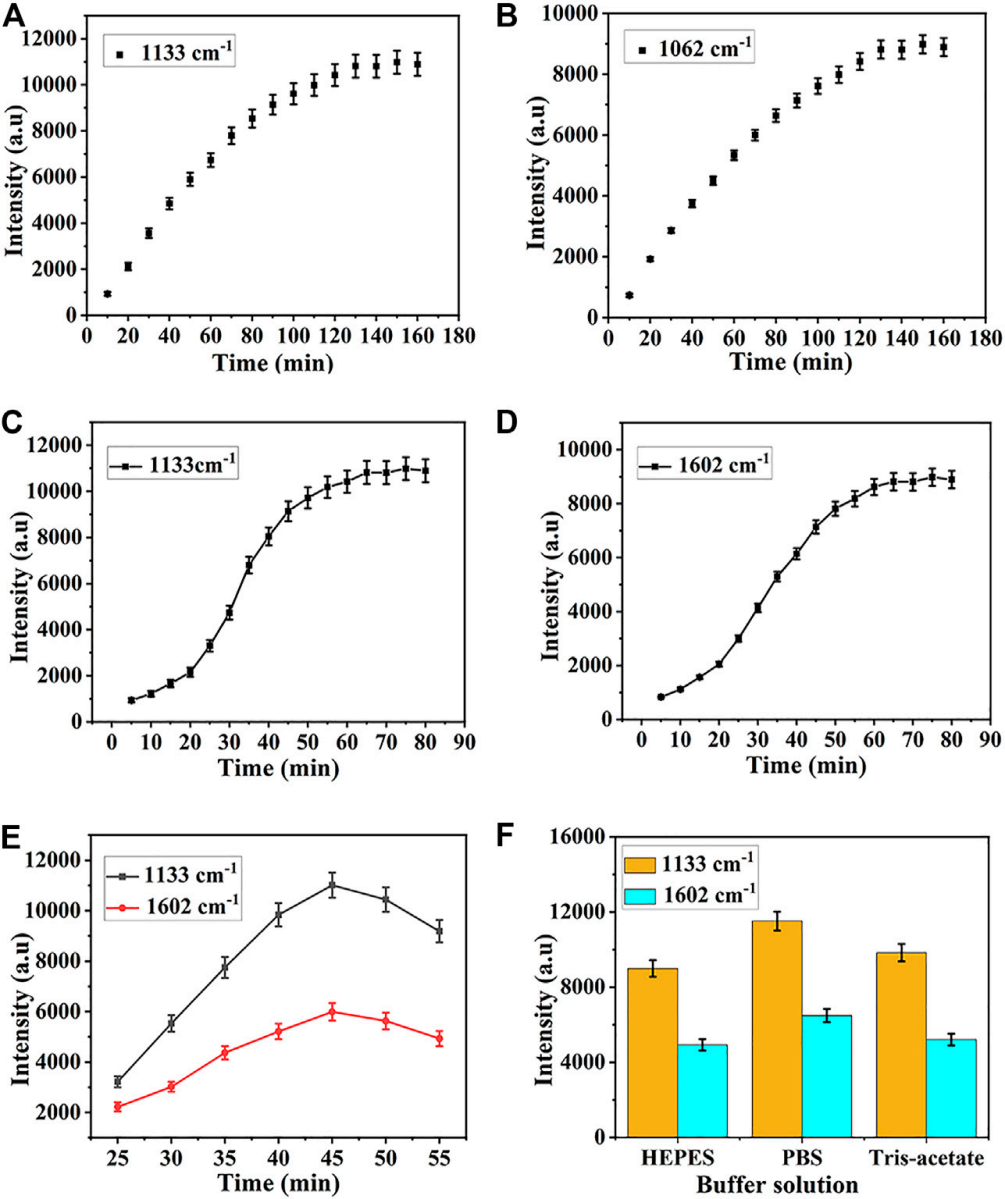
Optimization of **(A)** assembly time for HP1, **(B)** assembly time for HP2 **(C)** hybridization time for HP1, **(D)** hybridization time for HP2, **(E)** temperature, and **(F)** category of buffer solution.

Several key experimental parameters, including assembly time, hybridization time, incubation temperature, and type of buffer resolution were optimized to achieve the best detection results. The optimization procedure was carried out by modifying one parameter while holding the others. In these experiments, the characteristic peak of 5-FAM and Cy5 at 1,133 cm^−1^ and 1,602 cm^−1^ were employed to quantify the Raman intensity of miR-122-5p and miR-140-5p, respectively. Since the assembly time of HP1 and HP2 determined their assembly amount on the SERS substrate surface, it was necessary to optimize the assembly time to ensure the maximum amount. In Figure 5A, the intensity of the characteristic peak at 1,133 cm^−1^ was set as the time function, when the time was less than 60 min, the intensity of the peak at 1,133 cm^−1^ increased almost linearly with time. Then, the rising speed gradually slowed down and reached saturation at 130 min, indicating that the assembly amount of HP1 on the substrate surface reached a maximum, therefore, the optimal assembly time was 130 min for HP1. Similarly, the intensity of the peak at 1,602 cm^−1^ attained a steady state at 130 min (Figure 5B). The hybridization time of SERS biosensor to target miRNAs was critical for subsequent SERS analysis. Dropped the target miRNA onto the prepared SERS substrate, and placed it in a 37°C incubator for hybridization. The SERS detection was carried out at 10 min intervals. For each detection, 5 points were randomly selected in the test area and the average value of their SERS spectra was taken as the detection result. Finally, the optimal hybridization time was obtained according to the Raman intensity. As shown in Figure 5C, the intensity of the characteristic peak at 1,133 cm^−1^ increased with time and finally stabilized at 65 min. Therefore, the optimal hybridization time between miR-122-5p and HP1 was set to 65 min. Similarly, the maximum and stable SERS signal representing hybridization between HP2 and miR-140-5p was obtained when the hybridization time reached 65 min (Figure 5D). It can be seen from Figure 5E that with a rise in temperature ranging from 25°C to 45°C, the Raman intensity increased correspondingly, and after that, an opposite tendency can be observed with an increase in temperature. Therefore, 45°C was selected as the optimal temperature for subsequent experiments. Because the type of buffer solution also has significant impacts on hybridization efficiency, three commonly used buffer solutions, such as Tris-Acetate, PBS, and HEPES, were tested to select the optimal buffer solution. The results illustrated that the highest hybridization efficiency was found in PBS (Figure 5F). Therefore, PBS was selected as the buffer solution in the experiment.

### 3.6 Selectivity of the SERS biosensor

**FIGURE 6:**
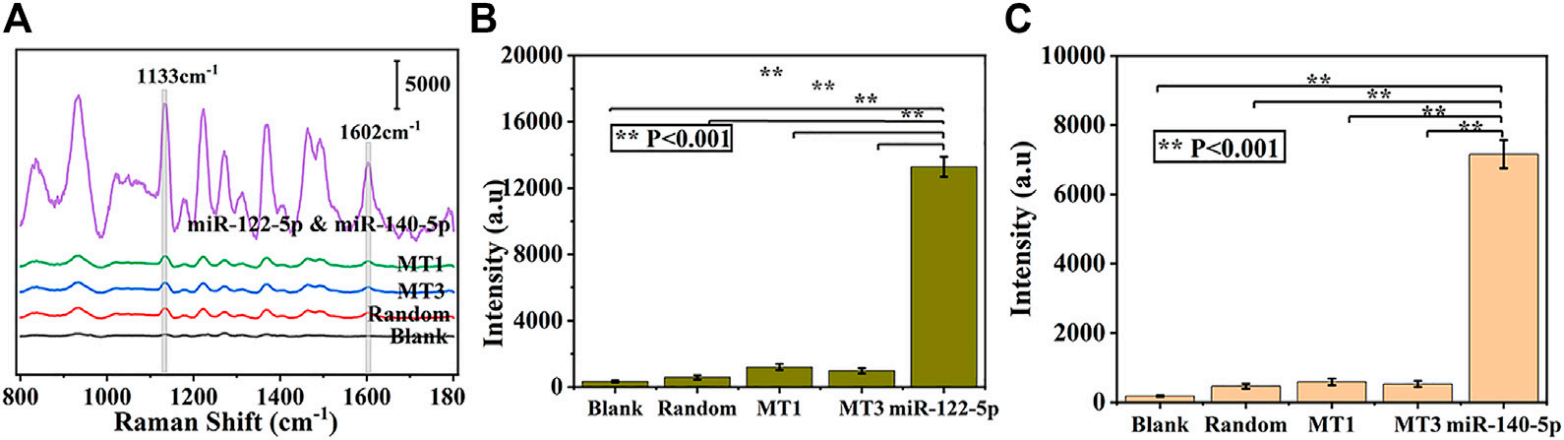
Specificity of the proposed biosensor, **(A)** and corresponding histogram of SERS intensities at 1,133 cm^−1^ **(B)** and 1,602 cm^−1^ **(C)** from three interference and blank control groups, respectively.

The selectivity of prefabricated SERS biosensors is considered to be a critical factor for practical applications. To assess the specificity of this strategy for simultaneous detection of miR-122-5p and miR-140-5p, several interference groups including single base mismatch sequences (MT1), triple base mismatch sequences (MT3), and random sequences were introduced. As described in Figures 6A–C, samples containing the same concentration of target miRNAs can effectively increase the intensity of the peaks at 1,133 cm^−1^ and 1,602 cm^−1^ compared with the interference and blank control groups, showing that the developed SERS biosensor has good selectivity and can tolerate the complex sensing environment.

### 3.7 Quantitative assays of miR-122-5p and miR-140-5p simultaneously

**FIGURE 7:**
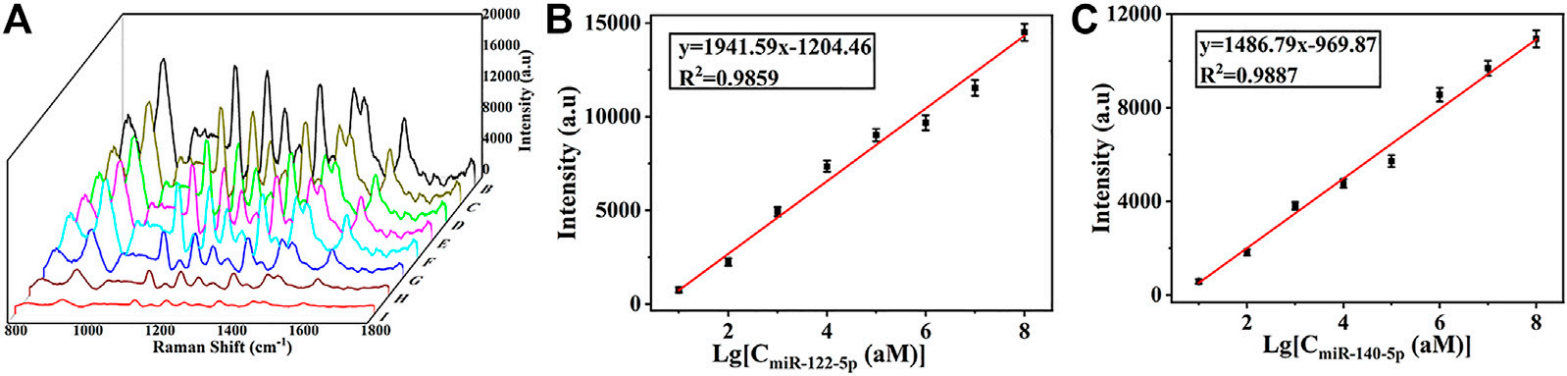
**(A)** SERS spectra of miR-122-5p and miR-140-5p with different concentrations in serum (10 aM, 100 aM, 1 fM, 10 fM, 100 fM, 1 pM, 10 pM, and 100 pM). **(B)** Calibration curve of peak intensities at 1,133 cm^−1^ *versus* logarithm of miR-122-5p concentration and **(C)** calibration curve of peak intensities at 1,602 cm^−1^ *versus* logarithm of miR-40-5p concentration.

A broad dynamic range and a low LOD were necessary for SERS biosensors to accommodate the extremely low concentrations of miRNAs in circulation. For quantitative miRNAs detection, eight samples were dropped onto the SERS substrate containing miR122-5p and miR140-5p at final concentrations of 10 amol/L, 100 amol/L, 1 fmol/L, 10 fmol/L, 100 fmol/L, 1 pmol/L, 10 pmol/L and 100 pmol/L in serum, respectively. SERS biosensors were hybridized with miRNAs of different concentrations in an incubator at 37°C for 65 min, then biosensors were taken out and washed with PBS buffer for SERS detection. In Figure 7A, a gradual increase in SERS intensity was observed with increasing miRNAs concentration. Furthermore, there was an excellent linear relationship between the SERS intensity and the logarithm of target miRNAs concentration (from 10 aM to 100 pM) (Figure 7B). The corresponding regression equation was y = 1941.59x-1204.46 (*R* ^2^ = 0.9859), where y is the SERS intensity at 1,133 cm^−1^, and the logarithm of miR-122-5p concentration is x. Similarly, the regression equation for miR-140-5p is y = 1,486.79x-969.87 (*R* ^2^ = 0.9887). The LOD was calculated using the formula: LOD = 3× (σ/S), where σ is the standard deviation of y-intercepts and S is the slope from the calibration curve. Thus, the calculated LOD for miR122-5p and miR140-5p were 4.17 aM and 4.49 aM, which is much more sensitive than some other highly sensitive detection methods. (Supplementary Table S3). Meanwhile, the detection time was shorter than most detection strategies. The results showed that the SERS sensor can quantitatively analyze the trace amounts of miR-122-5p and miR-140-5p in serum, meeting the practical needs of clinical diagnosis.

### 3.8 SERS biosensor in clinical application and accuracy evaluation

**FIGURE 8:**
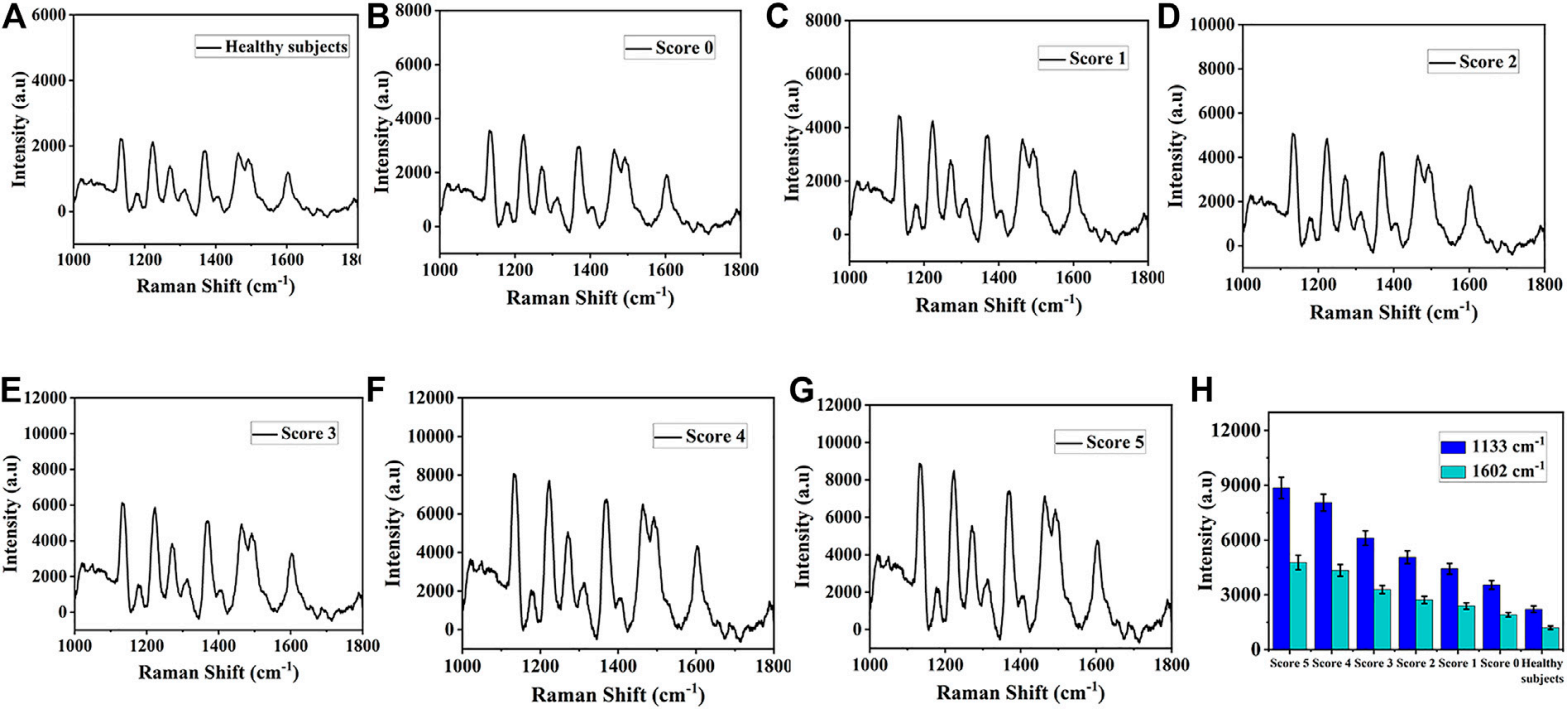
The average SERS spectra of miR-122-5p and miR-140-5p in serum obtained from **(A)** Healthy subjects **(B)** Score 0, **(C)** Score 1, **(D)** Score 2, **(E)** Score 3, **(F)** Score 4, and **(G)** Score 5. **(H)** Corresponding SERS intensities at 1,133 cm^−1^ and 1,602 cm^−1^.

In order to further verify the practicability of this SERS platform in real biological samples, the expression levels of miR-122-5p and miR-140-5p in peripheral blood of 20 healthy subjects and 40 patients with ICH at different scores (according to Hemphill score) were detected using SERS. Figures 8A–E showed the average SERS spectra of miR-122-5p and miR-140-5p in the blood of 20 healthy subjects and 40 ICH patients. The concentrations of miR-122-5p and miR-140-5p can be obtained by substituting the intensities of the characteristic peaks at 1,133 cm^−1^ and 1,602 cm^−1^ into the linear regression equation (Figure 7B), and the corresponding SERS intensities at 1,133 cm^−1^ and 1,602 cm^−1^ are shown in Figure 8F. It can be seen that the SERS signal intensities of miR-122-5p and miR-140-5p in ICH patients were significantly higher than those in healthy subjects. qRT-PCR was performed to verify the reliability of the SERS results, and the obtained results from both methods are presented in Supplementary Table S4. The results obtained *via* our SERS biosensor were in good concordance with those of qRT-PCR, demonstrating the high accuracy of the SERS biosensor platform for the simultaneous detection of miR-122-5p and miR-140-5p in biological samples.

## 4 Conclusion

In conclusion, a rapid, simple, and ultra-sensitive SERS biosensor was constructed for the simultaneous detection of two ferroptosis-related miRNAs in peripheral blood. The SERS biosensor was composed of DS Au nanorods-modified h-paper with HPs, which were hybridized with PHs and labelled with Raman reporters. The SERS substrate exhibited high specificity, good uniformity as well as excellent reproducibility, with LOD of 4.17 aM and 4.49 aM for miR-122-5p and miR-140-5p in serum, respectively. Moreover, by optimizing several key experimental parameters, such as assembly time, hybridization time, incubation temperature, and type of buffer resolution, the best performance of the SERS biosensor platform was achieved. Finally, the SERS biosensor and traditional qRT-PCR were employed to measure the expression of miR-122-5p and miR-140-5p in ICH patients and healthy subjects, and the results revealed that ICH patients had significantly higher levels of miR-122-5p and miR-140-5p than healthy subjects, which was supported by the qRT-PCR technique. Therefore, the proposed SERS biosensor can be used as a tool to detect miRNAs associated with ferroptosis following ICH in clinical samples, with potential clinical applications.

## Data Availability

The datasets presented in this study can be found in online repositories. The names of the repository/repositories and accession number(s) can be found in the article/Supplementary Material.
