# Supplementary material for: A simple and efficient strategy for trace detection of ferroptosis-related miRNAs based on novel hydrophobic paper-based plasmonic substrate and “inverse molecular sentinel (iMS)” nanoprobes
Source: Front Bioeng Biotechnol. 2023 Mar 2;11:1146111. doi: 10.3389/fbioe.2023.1146111 (PMC10017978; doi:10.3389/fbioe.2023.1146111)
Supplement: Supplementary file 1 [file DataSheet1.docx]

Supplementary Material

Rapid and ultra-sensitive detection of ferroptosis-related miRNAs based on novel dumbbell-shaped gold nanorods array and “inverse Molecular Sentinel (iMS)” nanoprobes

Youwei Wang^1,2^, Bing Chen^3^, Jiang Fan^1^, and Zhong Wang^1,4*^

*** Correspondence:** Zhong Wang, Department of neurosurgery& Brain and Nerve Research Laboratory, The First Affiliated Hospital of Soochow University, Suzhou, Jiangsu Province, 215006, China. E-mail: wangzhong761@163.com

# Supplementary Figures


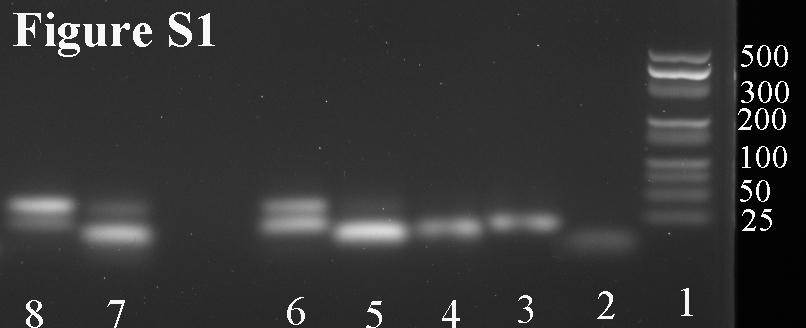


**Figure S1**: PAGE of iMS process (miR-122-5p). Lane 1: Marker; Lane 2: miR-122-5p; Lane 3: HP1; Lane 4: PH1; Lane 5: miR-122-5p+HP1; Lane 6: HP1+ PH1; Lane 7: miR-122-5p+HP1@ PH1. Lane 8: HP1+ PH1.


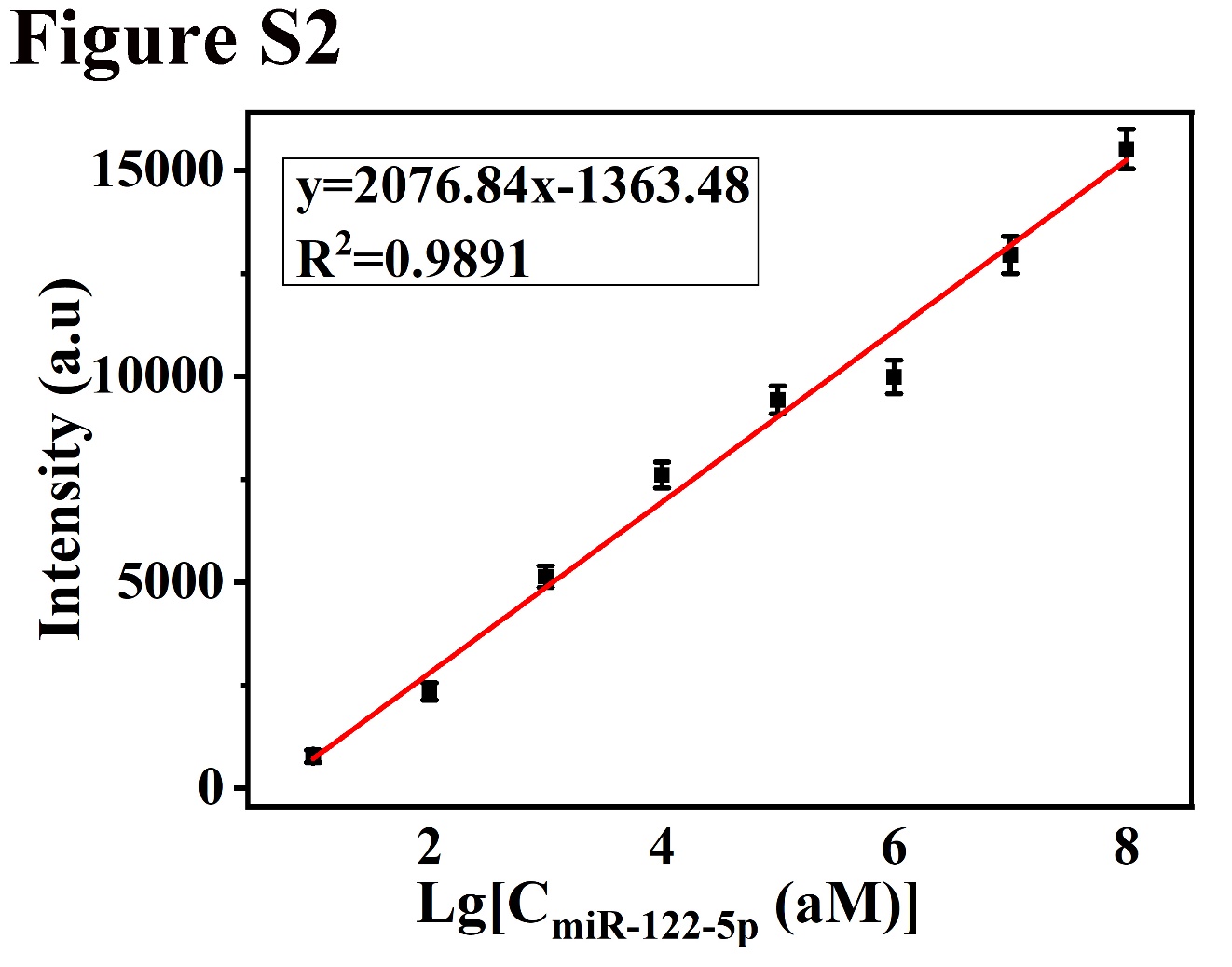


**Figure S2**: SERS spectra of miR-122-5p with different concentrations in PBS buffer (10 aM, 100 aM, 1 fM, 10 fM, 100 fM, 1 pM, 10 pM and 100 pM). Calibration curve of peak intensities at 1133 cm^-1^ versus logarithm of miR-122-5p concentration.

# Supplementary Tables

**Table S1** Sequences of oligonucleotides used in the experiment

| Name | Sequences (5'-3') |
| --- | --- |
| HP1 | HS-TCTACAATGGTGTTTGATGATTGTGTG-Cy5 |
| HP2 | HS-TCTTACCCTATGGTAGAAAGGGTATTG-5-FAM |
| PH1 | CCCTCCAAACACCATTGTCACACTCCA |
| PH2 | CCAACCTACCATAGGGTAAACCACTG |
| MT1-1 | UGGAGUGUCACAAUGGUGUUUG |
| MT1-2 | CAGUGGUUUUTCCCUAUGGUAG |
| MT3-1 | UGCAGUGUAACAAUGTUGUUUG |
| MT3-2 | CGGUGGUUUUCCCCUTUGGUAG |
| Random | ACCAGCUCGAGUAAGGAAAUG |
| miR-122-5p | UGGAGUGUGACAAUGGUGUUUG |
| miR-140-5p | CAGUGGUUUUACCCUAUGGUAG |

**Table S2** Basic characteristics of the participants in this study

| Groups | Healthy subjects | Score 0 | Score 1 | Score 2 | Score 3 | Score 4 | Score 5 |
| --- | --- | --- | --- | --- | --- | --- | --- |
| Average age | 57 | 60 | 58 | 57 | 63 | 65 | 66 |
| Gender |  |  |  |  |  |  |  |
| Male | 14 | 5 | 5 | 4 | 2 | 3 | 1 |
| Female | 6 | 5 | 3 | 4 | 4 | 2 | 2 |
| Sample number | 20 | 10 | 8 | 8 | 6 | 5 | 3 |

**Table S3** Comparison of the SERS biosensor with other miRNA detection strategies

| Method | Detection target | Linear range | LOD | Ref. |
| --- | --- | --- | --- | --- |
| Electrochemistry | miR-155 | 10 fM-10 nM | 39.6 aM | [43] |
| Fluorescence | miR-21 | 1 pM-1 nM | 0.05 pM | [44] |
| SERS | miR-203 | 10 fM-100 nM | 6.3 fM | [45] |
| SERS | miR-141 | 10 fM-10 nM | 2.92 fM | [46] |
| SERS | miR-122-5p | 10 aM-100 pM | 4.17 aM | This work |
|  | miR-140-5p |  | 4.49 aM |  |

**Table S4** Results of SERS and qRT-PCR in clinical serum samples

| Sample | SERS | | qRT-PCR | | Relative error (%) | |
| --- | --- | --- | --- | --- | --- | --- |
|  | miR-122-5p | miR-140-5p | miR-122-5p | miR-140-5p | miR-122-5p | miR-140-5p |
| Healthy subjects | 57.5 aM | 28.5 aM | 55.2 aM | 27.1 aM | 4. 17 | 5.41 |
| Score 0 | 0.28 fM | 86.5 aM | 0.27 fM | 82.8 aM | 3.70 | 4.37 |
| Score 1 | 0.79 fM | 0.18 fM | 0.75 fM | 0.17 fM | 5.33 | 5.19 |
| Score 2 | 1.68 fM | 0.31 fM | 1.62 fM | 0.32 fM | 3.70 | -3.18 |
| Score 3 | 5.75 fM | 0.74 fM | 5.97 fM | 0.70 fM | -3.69 | 5.70 |
| Score 4 | 58.88 fM | 3.7 fM | 55.49 fM | 3.6 fM | 6.11 | 2.78 |
| Score 5 | 151.3 fM | 7.4 fM | 144.2 fM | 6.9 fM | 4.93 | 7.24 |
